# Distribution of glucose transporters in renal diseases

**DOI:** 10.1186/s12929-017-0371-7

**Published:** 2017-08-31

**Authors:** Leszek Szablewski

**Affiliations:** Medical University of Warsaw, Chair & Department of General Biology & Parasitology, Center for Biostructure Research, 5 Chalubinskiego Str., 02-004 Warsaw, Poland

**Keywords:** Kidney, GLUT proteins, SGLT proteins, Diabetes, Familial renal glucosuria, Fanconi-Bickel syndrome, Renal cancers

## Abstract

Kidneys play an important role in glucose homeostasis. Renal gluconeogenesis prevents hypoglycemia by releasing glucose into the blood stream. Glucose homeostasis is also due, in part, to reabsorption and excretion of hexose in the kidney.

Lipid bilayer of plasma membrane is impermeable for glucose, which is hydrophilic and soluble in water. Therefore, transport of glucose across the plasma membrane depends on carrier proteins expressed in the plasma membrane. In humans, there are three families of glucose transporters: GLUT proteins, sodium-dependent glucose transporters (SGLTs) and SWEET. In kidney, only GLUTs and SGLTs protein are expressed. Mutations within genes that code these proteins lead to different renal disorders and diseases. However, diseases, not only renal, such as diabetes, may damage expression and function of renal glucose transporters.

## Background

Maintenance of glucose homeostasis prevents pathological consequences due to prolonged hyperglycemia or hypoglycemia. Hyperglycemia leads to a high risk of vascular complications, nephropathy, neuropathy and retinopathy. Hypoglycemia may damage the central nervous system and lead to a higher risk of death. Mechanisms of glucose homeostasis include glucose absorption in the gastrointestinal tract, glucose uptake in muscle and adipose tissue, gluconeogenesis in the liver, glucose reabsorption, and excretion in the liver and kidneys [1]. In the postabsorptive state, kidneys release approximately 20% of the total body glucose, are responsible for approximately 40% gluconeogenesis, and release for approximately half of all circulatory glucose during the fasting state [2]. Not only renal gluconeogenesis plays an important role in glucose release the kidneys filter and reabsorb glucose as well. For example, the glomeruli filter approximately 180 g of D-glucose from plasma per day. Reabsorption of D-glucose is done by the glucose transporters. These proteins are expressed in cell membranes of proximal tubular cells [3]. Because glucose is hydrophilic and soluble in water, lipid bilayer of plasma membrane is impermeable for it. Therefore, transport of glucose into cells depends on carrier proteins that are present in the plasma membrane. In humans, there are three families of glucose transporters: GLUT proteins, encoded by *SLC2* genes; sodium-dependent glucose transporters (SGLT) encoded by *SLC5* genes; and SWEET, encoded by the *SLC50* gene [4]. Mutations within these genes lead to different renal disorders. However, diseases may disturb the expression and function of renal glucose transporters.

In this review, I describe the role of glucose transporters in renal physiology and renal diseases.

## Renal glucose transporters in health

The expression of glucose transporters is tissue-specific. Every cell expresses one or more glucose transporters. These transporters show specific and regulatory properties.

### Renal GLUT proteins

The family of GLUT proteins belongs to the Major Facilitator Superfamily (MFS) of membrane proteins [5]. These proteins are encoded by the solute-linked carrier family 2, subfamily A, and gene family *SLC2A.* GLUTs are uniporters. They facilitate the diffusion of substrates across cellular membranes along a concentration gradient [6–8]. Only HMIT (GLUT13) is an H^+^/myo-inositol symporter [9]. Human GLUT proteins transport several hexoses, myo-inositol [9], urate [10, 11], glucosamine [12], and ascorbate [13]. Human GLUTs are proteins composed of ~500 aa (amino acid) residues. These proteins comprise 12 transmembrane helices (TMHs) with both their NH_2_ and COOH terminals positioned in the cytoplasm, a single N-linked glycosylation and a large, central, cytoplasmic linker domain. The sequence homology shows 14–63% identity. Based on sequence similarity, GLUT1 proteins are categorized into three classes [14]. About 14 human facilitative glucose transporters are described: GLUT1 – GLUT12, GLUT14, and HMIT (GLUT13). In a healthy kidney, four GLUT proteins are expressed: GLUT2, GLUT5, GLUT9 and GLUT10.

GLUT2 was cloned in 1988 from rat and human liver and kidney cDNA libraries [15, 16]. It is coded by *SLC2A2* gene (chromosome location 3q26.1-q26.2) [17]. The gene is comprised of 11 exons and is about 31 kb in length [17]. GLUT2 is composed of 524 aa with a molecular weight of 58 kDa [18]. GLUT2 has a low affinity for glucose (Km ~ 17 mM), fructose (Km ~ 76 mM), galactose (Km ~ 92 mM) and mannose (Km ~ 125 mM). It is a very high-affinity transporter for glucosamine (Km ~ 0.8 mM) [14, 19, 20]. GLUT2 is present in the hepatocytes, intestines (in the basolateral membrane of the epithelial cells), β-cells of pancreas [21], in a small number in neurons in the central nervous system, astrocytes [22, 23], and in tanycytes [24]. It is also located in the kidney, on the basolateral membrane of the epithelial renal tubules [21, 25]. In the intestines GLUT2 is involved in the release of glucose absorbed by SGLT1 into the bloodstream. In the kidney, it participates in the release of glucose into the blood, reabsorbed by SGLT1 and SGLT2 [8, 20]. In hepatocytes, GLUT2 releases glucose synthesized by gluconeogenesis into the blood, and in the pancreatic β-cells it provides glucose-sensing functions for insulin secretion [20]. In the brain, GLUT2 may participate in the glucose-sensing mechanism involved in control feeding and energy homeostasis [14].

GLUT5, the next member of GLUT proteins family was initially cloned from human small intestines [26]. It belongs to the class II GLUT proteins. GLUT5 is encoded by *SLC2A5* gene localized on chromosome 1p36.2 [14, 17]. The gene consists of 12 exons and is about 33 kb in length [17]. GLUT5 is composed of 501 amino acids with a molecular weight of approximately 43 kDa [18, 27]. GLUT5 is the sole transporter specifically for fructose and exhibits no transport activity for glucose or galactose. It mediates fructose transport with a Km ~ 6 mM [28]. GLUT5 is expressed at high levels in the jejunal region of the small intestine. It is also expressed at lower levels in the kidney, fat, skeletal muscles, brain, as well as at high levels in the spermatozoa of certain species [14, 18, 27, 29]. In the jejunum, it is localized to both the apical and basolateral membranes of the epithelial cells [30, 31]. GLUT5 mediates the uptake of fructose across the apical membrane of the small intestine, and then hexose is released into the bloodstream (into the portal vein) via GLUT2 localized in the intestinal basolateral membrane [8, 14].

In the kidney, GLUT5 is expressed in the apical plasma membrane of S3 proximal tubule cells [32]. Here, it can recapture fructose lost from glomerular filtration [33]. In the kidney of rats, GLUT5 has a V_max_ of 106 pmol/s per mg of protein and K_m_ of 12.6 mM [34]. Physiological concentration of fructose in the blood, and presumably glomerular filtrate, is ~0.008–0.03 mM. The concentration of fructose in the urine of healthy humans is 0.035 mM [33]. These differences in fructose concentrations determine the direction of fructose flux in which participates of GLUT5. In the basolateral membrane of proximal tubular cells, GLUT2 is also expressed and participates in the release of fructose, reabsorbed by GLUT5, into the blood.

GLUT9 was cloned from human cancer tissues [35]. The human *SLC2A9* gene is localized on chromosome 4p16-p15.3, contains 12–13 of exons and is approximately 214 bp in length [17]. GLUT9 belongs to the class II of GLUTs family. The human *SLC2A9* encodes two isoforms using alternative promoters. Human GLUT9a (the major isoform of GLUT9), encoded by 12 exons, is 540 aa in length. GLUT9b (GLUT9ΔN), encoded by 13 exons, is comprised of 512. These isoforms of GLUT9 protein differ only in their N terminus [14, 17]. In humans, GLUT9a is expressed in liver, kidney, intestine, leukocytes, and chondrocytes [14, 36, 37]. GLUT9b is expressed in the liver and kidney [36]. In kidney, GLUT9 is expressed in the proximal tubule [37]. In the epithelial cells, GLUT9a is present in the basolateral membrane, whereas GLUT9b is localized on the apical pole [37]. GLUT9 is a high-affinity transporter for glucose (Km = 0.6 mM) and fructose (Km = 0.4 mM). GLUT9 (both GLUT9a and GLUT9b) has been also identified as a high-affinity uric acid transporter (Km = 0.9 mM) [8, 14].

GLUT10 belongs to the class III of GLUT proteins. It has been cloned from human liver cDNA [38]. The human gene *SLC2A10* is localized on chromosome 20q13.1, contains five exons, and is approximately 27 bp in length [17, 39]. The gene codes for a 541-aa protein [17]. GLUT10 is expressed at highest levels in the liver and pancreas, and at lower levels in the heart, lung, brain, skeletal muscles, placenta, and in the kidney [38, 40]. GLUT10 shows a very high affinity for both deoxy-D-glucose (Km ~ 0.3 mM) and D-galactose [40], but not for fructose [41].

### Renal sodium-dependent glucose transporters

In 1960 Crane proposed the model of active, ATP-dependent glucose transport against a concentration gradient [42]. He developed the model of a mobile carrier in the brush border membrane (BBM) with two binding sites, one for glucose and one for ATP [43]. According to his hypothesis, the continuously outward Na^+^ ions gradient accomplished by the Na^+^/K^+^ ATPase on the basolateral membrane was the primary asymmetry providing the driving force for active sugar transport. It was considered by the “secondary active transport” (indirect active transport), as the hydrolysis of ATP was indirectly coupled to glucose transport via electrochemical gradient.

The *SLC5* family is the second family of glucose transporters. There are more than 220 members of this family. These transport proteins are also known as the sodium substrate symporter family (SSSF) [44]. In the human genome, 12 of these have been identified [4]. Sodium-glucose transporters are also known as Na^+^/glucose cotransporters or symporters (SGLTs). Human *SLC5* genes code for highly glycosylated membrane proteins of 580–718 aa residues, with a predicted mass of 73 kDa (60–80 kDa). The obtained results suggest the presence of 14 TMHs with both N- and C-terminal domains lying in the extracellular (luminal) area of the cell [45]. The organization of human *SLC5* genes is quite similar however, diversity in the gene structure is observed. For example, eight genes contain of 14–15 exons, one gene of eight exons, and one gene contains one exon. An alternative splicing is observed in the case of four *SLC5* genes [4]. These proteins are sodium cotransporters, except for SGLT3, encoded by *SLC5A4* gene, which is not a transporter but a glucosensor [46]. Sodium cotransporters transport different substrates such as monosaccharides (glucose, galactose, mannose, and fructose), vitamins (biotin, lipoate, and pantothenate), ions (Na^+^, I^+^, ClO_4_^−^, SCN^−^, NO_3_^−^, Br^−^), short chain fatty acids, myo-inositol and chiro-inositol, choline, as well as urea and water [4]. In a human kidney, nine sodium cotransporters are expressed: SGLT1, SGLT2, SGLT3, SGLT4, SGLT5, SGLT6, SMIT1, SMVT, and SMCT1 [4, 44].

SGLT1 was cloned in 1987 from rabbit intestinal mRNA [45]. It is an archetype for the *SLC5* family [47]. SGLT1 is encoded by gene *SLC5A1* localized on human chromosome 22q12.3 [4]. The gene is comprised of 15 exons, spanning 72 kb [48] or 67 kb, according to Zhao and Keating [17]. According to by Turk et al. [48], the gene originates from a six-membrane-span ancestral precursor via gene duplication. The gene codes for a membrane protein of 664 aa protein with a predicted mass of 73 kDa [49], or according to Zhao and Keating [17], the *SLC5A1* gene codes for a 662 aa membrane protein with a predicted mass of 73 kDa. This glucose transporter shows no sequence homology to GLUT protein. Sodium ions and sugar have two separate pathways through SGLT1. Two sodium ions are transported along with each glucose molecule, and the turnover time for one complete cycle approaches 1000s^−1^ at 37 °C [4]. SGLT1 binds sodium ions, before it binds glucose, and the electrochemical Na^+^ gradient generated by the Na^+^/K^+^-ATPase, is the driving force for the symporter activity [44].

SGLT1 is predominantly expressed in the small intestines, trachea, brain, and prostate [17, 50]. In the human heart, this transporter is unexpectedly expressed in high amounts, approximately 10-fold higher as compared with that in the kidney [51]. SGLT1 is also expressed in the renal proximal straight tubules (termed S3), where it plays an important role [25]. It contributes to 10% of the glucose reabsorbed in the proximal tubule [1]. In this way SGLT1 prevents glucose loss in urine.

SGLT1 is a high-affinity, low-capacity glucose transporter in renal proximal tubules. It is a high-affinity transporter for D-glucose (Km ~ 0.4 mM) and D-galactose [52]. It does not transport fructose, mannose and xylose [27]. It has been demonstrated that SGLT1 can transport 264 water molecules, besides the sodium ions and glucose [53]. In humans, 1 mol of glucose absorbed per day in the intestine determines the absorption of up to four liters of water and therefore, it plays an important role in water absorption in the brush border membrane of enterocytes [49].

SGLT2 was cloned in 1992 from the human kidney cDNA [8]. This transporter is coded by gene *SLC5A2*, localized on human chromosome 16p11.2 [4]. The gene is comprised of 14 exons (spanning 8 kb) [17]. The gene codes for a 672 aa protein with a predicted mass of 73 kDa [17, 54], and the protein has 59% identities on SGLT1 [54]. It is highly expressed in the kidney cortex and localizes on the apical domain of epithelial cells of the proximal tubule (S1/S2 segments) [4, 8]. It is also found in the brain, liver, thyroid, muscle, and heart [44, 50]. SGLT2 has been identified as a kidney-specific transporter. This transporter is responsible for reabsorption of 90% of glucose filtered at glomeruli [3]. It is responsible for the reabsorption of 180 g of glucose/day from the glomerular filtrate [4]. SGLT2 is a low-affinity (Km ≤ 6 mM), high-capacity glucose symporter in renal proximal tubules. It cannot transport D-galactose. The SGLT2 stoichiometry is 1 Na^+^: 1 sugar [55]. SGLT2 may also behave as glucose receptors in the heart and brain [49].

SGLT3. The human SGLT3 (SAAT1) cDNA was cloned from colon carcinoma [8]. It is encoded by gene *SLC5A4* localized on human chromosome 22q12.3 [4]. The organization of intron – exon is like *SLC5A1* [54]. The gene codes for a 660 aa protein [17]. SGLT3 stoichiometry is 2 Na^+^: 1 sugar. Human SGLT3 does not transport sugar. The sugar depolarizes the plasma membrane because glucose generates an inward Na^+^ current. Therefore, this transporter is a glucose sensor [17, 49, 56]. In humans, SGLT3 is expressed in cholinergic neurons in the enteric neurons system and at the neuromuscular junction [4]. SGLT3 may modulate action potentials in neurons/skeletal muscle cells glucose dependently [4, 8]. SGLT3 is also expressed in uterus, testis, lung, brain, thyroid and kidney [8, 50]. Little is known about the expression or activity of SGLT3 in the kidney [57]. SGLT3 is expressed in human proximal tubular cells [57]. In these cells, it might be responsible for sodium reabsorption [8, 57].

SGLT4. Little studies have been carried out with SGLT4. SGLT4 was cloned from human small intestine cDNA libraries [8]. It is encoded by *SLC5A9* gene localized on human chromosome 1p33. The gene codes for a 699 aa protein [44]. The internal splice in exon 14 adds either 38 or 53 aa between TMHs 13 and 14 [4]. SGLT4 is expressed in the kidney, liver, lung, brain, small intestines, heart, and uterus [4, 50]. It appears to transport, with a rank order, of mannose, glucose, fructose, and galactose. Results obtained by Tazawa et al. [58] suggest that SGLT4 is involved in mannose homeostasis. SGLT4 might be responsible for intestinal absorption and renal reabsorption of mannose.

SGLT5. As in the case of SGLT4, there are limited reports on the function of SGLT5. It was cloned from the human kidney cDNA [8]. The *SLC5A10* gene is localized on human chromosome 17p11.2 and codes for 596 aa protein [44]. There are different isoforms of this cotransporter, because exon 7 may be spliced out deleting 26 aa between TMHs 5 and 6, internal splice in exon 10 means either 36 or 52 aa between TMHs 11 and 12 [4]. It is expressed in the kidney cortex but its subcellular distribution and physiological role remains unknown. It has a relatively high affinity and capacity for mannose, therefore is treated as a kidney-specific sodium-dependent mannose transporter. SGLT5 also transports fructose, glucose and galactose [4, 8].

SGLT6, now known as SMIT2, was initially cloned from a rabbit kidney cDNA [8]. It is encoded by *SLC5A11* gene, localized on human chromosome 16p12 and codes for a 675 aa protein [50]. During the posttranscriptional process, splicing eliminates exon six and TMH four. SGLT6 is widely expressed in the human body: it is expressed in the brain, heart, skeletal muscle, spleen, liver, placenta, lung, leukocytes, and neurons [59]. It is also detected in the apical membranes of the rat intestine [60] and in the luminal side of the proximal convoluted tubules in the kidney of rabbits [61]. SGLT6 transports inositols but not glucose [62]. Human *SLC5A11* interacts with immune related genes and may function as an autoimmune modifier gene [63].

SMIT1, encoded by *SLC5A3* gene, was cloned from canine renal cells. The gene is localized on human chromosome 21q22.11 and codes for a 718 aa protein [4, 44]. There are three transcript variants due to splicing within, and distal to exon two: SMIT1a, SMIT1b, and SMIT1c. SMIT1b and SMICT1c lack the 14 TMH [4, 8]. It is expressed in the kidney, brain, placenta, pancreas, heart, skeletal muscles, and the lung [8]. SMIT1 is a Na^+^/myo-inositol cotransporter [64] (Km = 120 μM and 13 mM for inositol and sodium, respectively [65]). It also transports L-fucose and L-xylose, but not their D-isomers [8].

As in the case of the few previously described cotransporters, limited studies have been carried out with the next two sodium cotransporters, and their physiological role remains unknown.

SMVT, encoded by *SLC5A6* gene is localized on human chromosome 2p13, is a widely expressed multivitamin cotransporter. It is expressed in the brain, heart, kidney, lung, and placenta [4]. SMVT transports pantothenic acid, biotin, and α-lipoic acid. It was also found as a Na^+^/iodide cotransporter [66].

SMCT1, also known as AIT, is encoded by *SLC5A8* gene, localized on human chromosome 12q23.1. It is detected in the small intestine, kidney, brain, retina, and muscle [4]. Its predominant substrates are short chain fatty acids; however, it has a high affinity for lactate, and it transports pyruvate and nicotinate with a stoichiometry of 2: 1 [67, 68].

### SWEET proteins

This is a new class of glucose transporters, encoded by *SLC50* gene. This class was first identified by the expressing candidate *Arabidopsis thaliana* genes coding for membrane proteins. SWEET transporter is predicted to have seven TMHs [4]. In the human genome, there is a single homolog (SWEET1) of SWEETs, encoded by *SLC50A1* gene. Human SWEET1 does not cause the uptake of glucose, but mediates a weak efflux. It was found predominantly in the Golgi apparatus with a minimum expression in the plasma membrane. Human SWEET1 shows the highest level of expression in the oviduct, epididymis, and intestines; to date, no expression was found in the kidney [4].

## Renal glucose transporters in diseases

Expression of glucose transporters changes in different organs during diseases [69–72]. These disturbances also are observed in renal diseases.

### Diabetic kidney

Diabetes mellitus is a common chronic disease. It is characterized by long-lasting hyperglycemia, due to lack of pancreatic insulin secretion (type 1 diabetes mellitus) and/or insulin resistance in peripheral organs (type 2 diabetes mellitus). Diabetic renal disease occurs in approximately 30–35% of patients with both types of diabetes; and mortality in patients with diabetes renal disease is nearly 20–40 times higher than that in patients without nephropathy [73]. The typical long-term complications in both forms of diabetes is the disease of the kidney, nephropathy. Hyperglycemia is a key factor in the pathogenesis of diabetic nephropathy [74]. Nephropathy is one of the major microvascular complications of diabetes. The typical histological changes of diabetic nephropathy are present in the mesangial cells.

Diabetes mellitus causes chronic renal failure (CRF) that is associated with significant cardiovascular morbidity and mortality. CRF is related to diverse associations of carbohydrate and insulin metabolism. This disease is characterized by a loss of nephron units that induces, as a compensatory mechanism, the glomerular hyperfiltration, and tubular hypertrophy. These pathologies may lead to glomerular sclerosis and progression to the end-stage renal disease [75]. CRF affects approximately 13% of the U S population [76]. Diabetes mellitus also causes the end-stage renal disease (ESRD). The prevalence of some degree of renal involvement in patients with diabetes reaches 40% [77], with significant progression to the end-stage disease [78].

Glucose is filtered by the renal glomeruli. As mentioned earlier in the paper, glucose is reabsorbed by the sodium-dependent glucose cotransporters across the BBM of the proximal tubule, and then by facilitative glucose transporters, is returned to the circulation. There was observed a threefold increase in renal glucose reabsorption in patients with diabetes as compared with a healthy control [79]. Hyperglycemia in patients with diabetes increases the expression of glucose transporters in the proximal tubule. Therefore in these patients, the capacity for glucose reabsorption in the proximal tubule is increased. There are differences in the prevalence of glomerular hyperfiltration in patients depending on the type of diabetes. These values are 13–75% in patients with type 1 diabetes, and 0–40% in patients with type 2 diabetes [80].

In patients with diabetes changes in expression and activity of glucose transporters are observed. There are different observations on SGLT1 in kidney. In the animal models of diabetes, no changes in the expression of SGLT1 protein in the kidney were observed during long-lasting hyperglycemia [81]. However, in the obese Zucker rats, an increased expression of SGLT1 mRNA due to diabetes was observed [82]. There are controversial finding about activity and expression of SGLT2. Rahmoune et al. [83] have investigated the expression of glucose transporters in the human exfoliated proximal tubular epithelial cells (HEPTEC) isolated from fresh urine. Researchers have found that in these cells received from patients with type 2 diabetes, expression of SGLT2 was significantly higher in comparison with that in healthy subjects. These results were confirmed in animal studies. The experiments were performed on renal cortex and medulla samples from control rats, diabetic rats with glycosuria, as well as normal, low, and high Na^+^-diet fed rats [84]. In animal models of diabetes, an increased expression of SGLT2 mRNA was observed [82, 85]. Authors suggested that overexpression of SGLT2 causes the development of diabetic renal tubular and glomerular disease. It was found, that human hepatocyte nuclear factor-1α (HNF-1α), a transcriptional factor, that is expressed in liver, kidney, pancreas and intestine, directly controls expression of SGLT2 gene [86]. It is a regulator of glucose homeostasis. In HNF-1α-deficient animals, the transcription of the SGLT2 is affected. In these animals, renal proximal tubular reabsorption of glucose is affected, producing severe renal glucosuria [87]. In humans, mutations in HNF-1α gene cause MODY3 (Maturity Onset Diabetes of Young). Results obtained by Freitas et al. [85] in animal studies showed that diabetes increases both SGLT2 and HNF-1α mRNA expression (~50%). Of note, it was observed, that expression of SGLT2 mRNA and HNF-1α and activity correlate positively in kidney of diabetic rats. Researchers also showed that changes due to diabetes are reversed by lowering glycemia, independently of insulinemia. Therefore, authors postulate, that HNF-1α, as a modulator of SGLT2 expression, may be involved in diabetic kidney disease [85]. Other results were obtained in other investigations. Interesting experiment was performed on rats during streptozotocin-induced diabetes [88]. Researchers have found that activity of SGLT2 decreases at 3, 7, and 14 days after injection of STZ. Authors suggest that decreased activity of SGLT2 plays a protective role to control the excess of circulating glucose. Decreased activity of transporter may be due to decreased of expression of SGLT2 (days 3 and 7) and changes in membrane lipid composition (day 14) [88].

There was also demonstrated an increased renal glucose uptake in isolated cells of patients with type 2 diabetes as compared with those of healthy control [83]. Also, increased glucose transport was observed in patients with type 2 diabetes [89]. In animal models of diabetes, an increase in the expression of the GLUT2 in the renal proximal tubules [81, 90, 91] and translocation of this transporter to the luminal surface of the proximal tubular cell have been shown [91]. Also in the experiments with the HEPTECs isolated from the urine of patients with type 2 diabetes, an elevated level of GLUT2 was observed [83]. Of note is that circulating glucose concentrations influence the expression of GLUT2 at the proximal tubule brush border membrane. Genetic variation of GLUT2 may be the cause of diabetic nephropathy. Hyperglycemia induces mesangial cell damage. In patients with diabetes and in animal models of diabetes an elevated level of transforming growth factor-β (TGF-β) in the glomeruli has been shown. This cytokine induces the expression of GLUT1 mRNA [92]. The overexpression of GLUT1 elevates the intracellular glucose accumulation and the formation of extracellular matrix component such as fibronectin, collagen, and laminin [93]. However, decreased levels of GLUT1 protein and GLUT1 mRNA in animal models of diabetes was observed [84, 90]. Based on the obtained results, it is suggested that overexpression of GLUT1 in mesangial cells is a key event in the development of nephropathy in patients with diabetes [94, 95]. However, in STZ-induced diabetic animals, no change in GLUT1 levels were observed at the proximal tubule BBM [91]. Of note is that genetic variation of GLUT1 affects nephropathy and may be associated with the risk of micro- and macroalbuminuria in the adult European Americans with type 2 diabetes [96]. Diabetes also influences the expression of GLUT5, a fructose transporter. It was observed, in STZ-induced diabetic rats, levels of GLUT5 proteins and GLUT5 mRNA at the proximal tubule BBM increased [90, 91].

SGLT2 plays an important role in renal glucose reabsorption. As mentioned earlier, the prevalence of glomerular hyperfiltration is observed in patients with diabetes. The hyperfiltration leads to the death of the glomeruli. Therefore, there occurs a higher filtration rate in the remaining glomeruli. This pathology causes loss of more glomeruli and results in an end-stage renal disease [80]. Hyperfiltration is due to the overexpression of SGLT2 in a diabetic kidney. Therefore, inhibition of SGLT2 may protect human proximal tubular cells [97, 98] by lowering of glomerular hyperfiltration and by limiting hyperglycemic damage to proximal tubule cells [80]. It also increases urinary glucose excretion. Animal studies showed that inhibitors of SGLT2 reduce albuminuria and kidney growth. However, the clinical evidence is unclear [99]. Inhibitors of SGLT2 are also treated as drugs used in diabetes mellitus [100–104]. Different inhibitors of SGLT2 are used as a novel treatment for patients with diabetes [105–108]. But the long-term effects of SGLT2 inhibitors are unknown. Therefore, clinical research remains to be carried out on the long-term effects of this class of drug.

In patients with diabetes, an enhanced proximal tubule sodium reabsorption has been observed [109]. An increased proximal tubule sodium reabsorption was confirmed in animal models of diabetes [110–112]. In humans proximal tubular cells express SGLT3 [57]. This cotransporter does not transport glucose. It facilitates influx of Na^+^ in the presence of extracellular glucose [56]. Results obtained by experiments performed on COS-7 cells and HK-2 cells (mammalian kidney-derived cells) showed that upregulation of SGLT3 in these cells increases intracellular sodium concentration by 3-folds without affecting glucose transport, and activation of SGLT3 increases sodium uptake in HK-2 cells by 5.5-fold [57]. The obtained results suggest that in diabetic models of animals, SGLT3 is overexpressed in the proximal tubule. Therefore, it is suggested that SGLT3 plays a role in enhancing proximal tubule sodium absorption and therefore promotes hyperfiltration and renal injury [57]. A predominant role in the reabsorption of Na^+^ in renal proximal tubule plays Na^+^/H^+^ exchanger 3 (NHE3). It plays also a major role in bicarbonate reabsorption in renal tubules [113]. However, the role of tubular NHE3 in the diabetic kidney remains incompletely understood [114]. Because NHE3 is electro-neutral, it is beneficial as the path for Na^+^ reabsorption. SGLTs are electrogenic [45], so that they depolarize the membrane, which is the disadvantage for SGLTs to be used as a paths for Na^+^ reabsorption. Increased Na^+^ retention by the proximal tubule NHE3, due to increased NHE3 activity, can play a role in some forms of hypertension observed in patients with diabetic kidney disease. There are also reports in humans that show increased proximal tubular Na^+^ reabsorption in patients with diabetes mellitus. It was found in children with Type 1 diabetes a significant increase (~20%) in proximal tubular reabsorption as determined by fractional lithium clearance [115] and in adults with Type 2 diabetes also found a ~ 20% change in reabsorption rates [116].

### Inhibitors of SGLT2 as anti-hyperglycemic agents

As described earlier, SGLT2 reabsorbs about 90% of glucose filtered in glomeruli [3]. On this way is reabsorbed of 180 g of glucose per day from the glomerular filtrate [4].

It was observed in the late 1980s that the administration of phlorizin in animal models of type 2 diabetes, induces glucosuria, and normalizes both fasting and fed plasma glucose levels [106, 117]. Of note, phlorizin was isolated in 1836 from bark of apple tree [118].

The US Food and Drug Administration has approved 3 inhibitors of SGLT2 for treatment of type 2 diabetes: canagliflozin, dapagliflozin and empagliflozin [104, 106]. In Japan have been approved luseogliflozin, topogliflozin and ipragliflozin [104]. Inhibitors of SGLT2 can be used as monotherapy or in combination with other oral agents as well as with insulin [104]. Canagliflozin acts also as a SGLT1 inhibitor [106]. Inhibitors of SGLT2 act on the kidney without adverse gastrointestinal effect [106]. The action of inhibitors is independent of insulin secretion, therefore the risk of hypoglycemia is low. Recently, these agents are not approved in type 1 diabetes [104]. Of note, it was shown in animal studies, that in normal animals, inhibitors of SGLT2 have no effect on plasma glucose levels. In this case, liver compensates glycosuria by the increase glucose synthesis. It is suggested, on the basis of animal studies, that inhibitors prevent of glomerular hyperfiltration, reduces albuminuria, kidney growth, and attenuated of inflammation.

In healthy subjects all the filtered glucose is reabsorbed in the proximal tubule. Therefore glucose is absent in urine. In patients with type 2 diabetes, inhibitors of SGLT2 stimulate glucose excretion through the kidney. The inhibitors increase glucose excretion rate of ~80 g/day [105]. Therefore produce glycosutia and reduce plasma glucose concentrations [103]. This is due to reduction of glomerular hyperfiltration. It was also observe that inhibitors of SGLT2 decrease of HbA1c (~ 1%) [105], lower blood pressure (~ 5 mmHg) [119] and cause weight loss (1,0–3,0 kg) [80]. On the other hand, there are unknown long-term effects of SGLT2 inhibitors. It was found, for example, an increase in the occurrence of urogenital infections in women after administration of inhibitors of SGLT2 [105, 107].

### Familial Renal Glycosuria

Familial renal glycosuria (FRG) is a rare renal tubular disorder occurring due to autosomal recessive mutations in the *SLC5A2* gene, characterized by the decreased reabsorption of glucose and therefore glucose is excreted through urine. It does not affect other glomerular tubular kidney functions [120]. Glucose excretion ranges from 1 to 162 g·1.73 m^−2^ ·day^−1^ [42]. In case of a complete absence of glucose reabsorption from the glomerular filtrate, the level of excretion is >160 g/day [121]. Forty-nine different mutations scattered throughout the *SLC5A2* gene have been reported, most of these mutations being private [122, 123]. There are missense and nonsense mutations (premature stop), small deletions (in-frame and frameshift) and splicing mutations. Results obtained by Santer et al. [121] and Calade et al. [123] showed that intron 7 is considered a mutational hot spot. IVS7 + 5G > A alleles were detected in several unrelated families of different ethnic regions, who exhibited FRG.

Only a small number of individuals present with polyuria and/or enuresis. Many heterozygous individuals for SGLT2 mutations, both nonsense and missense, suffer mild glycosuria, (<10 g/1.73m^2^/24 h) which is relatively common. Patients with severe glycosuria (≥10 g/1.73m^2^/24 h) show the recessive inheritance with homozygosity or compound heterozygosity for SGLT2 mutations [121, 123]. In type O glycosuria, extremely rare cases, the reabsorption of glucose is severely reduced or absent [100]. A reduced number of functioning SGLT2 proteins in the renal tubule, due to haploinsufficiency, leads to type A glycosuria. Decreased in SGLT2 affinity for glucose, due to missense mutations, leads to type B glycosuria [121]. FRG might be due to genetic heterogeneity – candidate genes, such as for example *GLYS1*, localized on human chromosome 6 might be a cause of FRG [124]. There were described patients with FRG with no mutations in the coding region of *SLC5A2* gene [121].

### Glucose–Galactose Malabsorption

Glucose–galactose malabsorption (GGM) was first described in 1962 as a severe watery diarrhea in newborn children [42]. These patients remain intolerant to glucose and galactose. GGM is a rare autosomal disease caused due to mutations within *SLC5A1* gene [8, 45, 122]. In this case, within the SGLT1 gene missense, nonsense, frame-shift, and specific-site mutations were described. Mutations may cause a failure to insert SGLT1 into the enterocyte and tubular membrane correctly [125], as well as the absence of the functioning SGLT1 within the apical plasma membrane [126]. Patients with GGM present mild or no renal glycosuria [53, 122]. Chronic dehydration might cause nephrolithiasis and nephrocalcinosis that develop in many cases [127]. Nephrocalcinosis may be caused by hypercalcemia, metabolic acidosis, and dehydration due to renal tubule dysfunction [45].

### Fanconi–Bickel syndrome

Fanconi–Bickel syndrome (FBS) is an extremely rare glycogen storage disease (GSD): only 112 GSD patients have been reported worldwide [8]. The first patient was reported in 1949 by Fanconi and Bickel [128]. This congenital defect is due to GLUT2 deficiency. FBS is an autosomal recessive disorder, and a total of 34 different GLUT2 mutations homozygous and heterozygous, have been described [8]. Patients with FBS, present at an age range 3–10 months, revealed hepatomegaly, glucose and galactose intolerance, a Fanconi-type nephropathy with severe glycosuria, and fasting hypoglycemia [129, 130]. The patients have a general impairment of tubular function. These patients accumulate free glucose and glycogen, which is due to the impairment of GLUT2 that facilitates exit of glucose and galactose at the basolateral membrane of renal tubular cells [122]. Because the transport of glucose out of renal tubular cells is an impairment, there occurs an accumulation of glycogen and free glucose within these cells. As an effect of this disturbance, an impairment of other transport functions resulting in a tubulopathy with disproportionately severe glycosuria occurs. In most patients, tubular glucose reabsorption is dramatically reduced or is even zero [129]. This may contribute to the development of hypoglycemia. Other effects of proximal tubular dysfunction are hyperaminoaciduria, hyperphosphaturia, hypercalciuria, renal tubular acidosis, mild tubular proteinuria, and polyuria.

### Renal cancers

Renal cancers affect nearly 270,000 patients annually worldwide. These cancers cause more than 115,000 deaths each year [131]. There are different types of kidney cancers, as for example von Hippel–Lindau (VHL), heredity papillary renal carcinoma (HPRC), Birt–Hogg–Dubé (BHD), hereditary leiomyomatosis renal cell carcinoma (HLRCC), succinate dehydrogenase kidney cancer (SHD-RCC), and so on [132]. Tumor cells have a dysregulated glucose metabolism. These cells have a reduced ability to use oxidative metabolism. Therefore, tumor cells increase the rate of glycolysis in the presence of oxygen, known as Warburg effect, and they have an increased use of glucose. An increased glycolytic metabolism increases the rate of glucose uptake [133]. Therefore, the dysregulation of glucose transporters’ expression has been described in malignant cells [134]. Hypoxia is a hallmark of cancer, changing GLUT expression [135]. It induces expression of hypoxia-inducible factor 1α (HIF-1α) that leads to downstream transcription of several genes, including *SLC2A1.* The VHL protein inactivates HIF-1α in physiological conditions. In many renal tumors, mutations of the VHL gene cause the synthesis of nonfunctional protein, leading to perpetuation of HIF-1α activity [136].

Renal cell carcinoma (RCC) is the most common malignancy arising in the adult kidney, which constitutes 2–3% of all malignant tumors in adults [137]. They are divided into different histological subtypes: clear cell carcinoma (ccRCC) that is the most common type (75–83% of cases), the papillary subtype (10–15% of cases), and the chromophobe subtype (5% cases) [138–140]. RCC influences the expression of GLUT proteins. The levels of expression of these transporters depend on both the type of RCC and the kind of GLUT protein. It was observed that in the case of ccRCC, GLUT1 was significantly upregulated as compared with healthy kidney tissue [137, 141, 142]. Increase of GLUT3 mRNA levels in these patients was also observed [143]. However, the expression of GLUT4, GLUT9, and GLUT12 was decreased in ccRCC [141]. These authors also observed that in chromophobes, RCC expression of GLUT4 is increased, whereas the GLUT2 and GLUT5 are downregulated. In the other subtype of RCC, in oncocytoma RCC, no changes were detected in the expression of investigated GLUT proteins, as compared with that in the normal healthy tissue [141]. Results obtained by other authors [143] showed that in patients with renal oncocytoma, the levels of GLUT1 mRNA were increased, whereas no changes were observed in patients with renal B-lymphoma. GLUT2 mRNA was markedly downregulated in patients with ccRCC, oncocytoma, and renal B-lymphoma [143].

There are also differences in levels of GLUT1 expression depending on the subtype of RCC. The higher expression of this transporter was observed in ccRCC in comparison with normal kidney tissue, chromophobe RCC, and papillary RCC [136, 144, 145]. No significant correlation between GLUT1 expression and tumor grade or tumor stage was found. However, authors have suggested that expression of GLUT1 may be a marker in the differential diagnosis and classification of renal tumors [136]. GLUT1 can be also a target-specific therapy as an anticancer therapy [136, 145]. In human RCC, GLUT5 is also upregulated. The expression of this transporter was higher in these specimens in comparison with that in chromophobe and papillary types [146]. GLUT5 is known as the transporter specific for fructose with no ability to transport glucose or galactose. These results suggest the other pathways for hexose metabolism in many RCCs. This pathway may be an additional source of energy for cancer. Based on the obtained results, authors postulate that GLUT5 may be correlated with grade II differentiation and may play a role in RCC development [146].

Interesting results were obtained by Chan et al. [142]. Authors investigated the correlation between the expression of GLUT1 and GLUT2 proteins depending on the presence or lack of VHL. In cells lacking VHL, GLUT1 was highly expressed, whereas in cells with VHL, very low levels of this protein were detected. However, in cells with VHL, the expression of GLUT2 was higher as compared with cells lacking VHL. As mentioned above, GLUT1 levels were high in RCCs, whereas GLUT2 levels were high in normal renal cells [142]. Hypoxia, as mentioned earlier, a specific state in tumors, induces expression of HIF-1α. This transcriptional factor regulates the expression of enzymes and other proteins involved in the glycolytic pathway, for example, it stimulates the expression of GLUT1 and GLUT3 [143]. In the physiological state, HIF-1α is inactivated by the VHL protein. The loss of nonfunctional VHL protein, due to mutations in tumor suppressor gene, occurs in about 80% of RCCs [142]. A significant correlation between the expression of GLUT1 and HIF-1α was also observed in patients with ccRCC [144].

An abundant glycogen-rich cytoplasm characterized ccRCC. This pathology is due to the aberrant influx and storage of glucose. There was found an association between GLUT1 gene polymorphism with ccRCC [147]. Therefore, authors suggest that *SLC2A1* is involved in ccRCC, increasing the susceptibility to the development of cancer however, its role is unclear.

### Urate metabolism disorders

A major regulator of urate homeostasis is GLUT9. It is a high-affinity uric acid transporter (Km = 0.9 mM). Urate is secreted into the blood stream from the liver by GLUT9 and is absorbed in the proximal convoluted tubule of the kidney. This mechanism regulates plasma levels of urate (250–300 μM) [148]. Urate is transported across the renal epithelium by URAT1, expressed in the apical membrane and by GLUT9a, present in the basolateral membrane. A significant association of GLUT9 expression with serum uric acid levels and with gout was reported [11]. Mutations in the *SLC2A9* gene may affect plasma uric acid levels. Hypouricemia is due to the loss of GLUT9 function. It is a consequence of both a reduced release of urate from the liver and disturbed renal reabsorption of urate from the urine [148–150]. Monogenic forms of hypouricemia have been linked with mutations in the SLC2A9 gene [11]. Loss of function of GLUT9 due to homozygous mutations cause a total defect of uric acid absorption. This defect causes severe hypouricemia, complicated by nephrolithiasis and exercise-induced acute renal failure [8]. However, increased expression of GLUT9 causes hyperuricemia and gout [8, 148]. Interestingly, in mice, in contrast to the condition in humans, loss of function of GLUT9 causes hyperuricemia, hyperuricosuria, and early-onset nephropathy [8, 11].

## Conclusions

Glucose transporters play an important role in renal functioning. These membrane transport proteins release glucose derived from renal gluconeogenesis. They also reabsorb glucose from urine. Disturbances in expression and/or function of glucose transporters cause renal diseases. However, renal diseases may cause impairment of glucose transporters.
